# Development and validation of a new knowledge, attitude, belief and practice questionnaire on leptospirosis in Malaysia

**DOI:** 10.1186/s12889-018-5234-y

**Published:** 2018-03-07

**Authors:** Wan Mohd Zahiruddin, Wan Nor Arifin, Shafei Mohd-Nazri, Surianti Sukeri, Idris Zawaha, Rahman Abu Bakar, Rukman Awang Hamat, Osman Malina, Tengku Zetty Maztura Tengku Jamaludin, Arumugam Pathman, Ab Rahman Mas-Harithulfadhli-Agus, Idris Norazlin, Binti Samsudin Suhailah, Siti Nor Sakinah Saudi, Nurul Munirah Abdullah, Noramira Nozmi, Abdul Wahab Zainuddin, Daud Aziah

**Affiliations:** 1 Department of Community Medicine, School of Medical Sciences, Universiti Sains Malaysia, 16150 Kubang Kerian, Kelantan Malaysia; 2 Unit of Biostatistics and Research Methodology, School of Medical Sciences, Universiti Sains Malaysia, Kubang Kerian, Kelantan Malaysia; 3 Ministry of Health Malaysia, Institute of Behavioural Health Research, Jalan Rumah Sakit Bangsar, Kuala Lumpur, Malaysia; 4 Department of Medical Microbiology and Parasitology, Faculty of Medicine and Health Sciences, Universiti Putra Malaysia, Serdang, Selangor Malaysia; 5 Disease Control Division, Complex E, Ministry of Health Malaysia, Putrajaya, Malaysia

**Keywords:** Leptospirosis, Knowledge, Attitude, Belief, Practice, Questionnaires, Development, Validation

## Abstract

**Background:**

In Malaysia, leptospirosis is considered an endemic disease, with sporadic outbreaks following rainy or flood seasons. The objective of this study was to develop and validate a new knowledge, attitude, belief and practice (KABP) questionnaire on leptospirosis for use in urban and rural populations in Malaysia.

**Methods:**

The questionnaire comprised development and validation stages. The development phase encompassed a literature review, expert panel review, focus-group testing, and evaluation. The validation phase consisted of exploratory and confirmatory parts to verify the psychometric properties of the questionnaire. A total of 214 and 759 participants were recruited from two Malaysian states, Kelantan and Selangor respectively, for the validation phase. The participants comprised urban and rural communities with a high reported incidence of leptospirosis. The knowledge section of the validation phase utilized item response theory (IRT) analysis. The attitude and belief sections utilized exploratory factor analysis (EFA) and confirmatory factor analysis (CFA).

**Results:**

The development phase resulted in a questionnaire that included four main sections: knowledge, attitude, belief, and practice. In the exploratory phase, as shown by the IRT analysis of knowledge about leptospirosis**,** the difficulty and discrimination values of the items were acceptable, with the exception of two items. Based on the EFA, the psychometric properties of the attitude, belief, and practice sections were poor. Thus, these sections were revised, and no further factor analysis of the practice section was conducted. In the confirmatory stage, the difficulty and discrimination values of the items in the knowledge section remained within the acceptable range. The CFA of the attitude section resulted in a good-fitting two-factor model. The CFA of the belief section retained low number of items, although the analysis resulted in a good fit in the final three-factor model.

**Conclusions:**

Based on the IRT analysis and factor analytic evidence, the knowledge and attitude sections of the KABP questionnaire on leptospirosis were psychometrically valid. However, the psychometric properties of the belief section were unsatisfactory, despite being revised after the initial validation study. Further development of this section is warranted in future studies.

## Background

Leptospirosis is considered a major re-emerging zoonosis of global and public health importance, particularly in developing countries due to socioeconomic conditions that favor human exposure and climatic conditions that favor endemicity in animal vectors [1]. There were more than 1 million clinical cases of leptospirosis occur annually, and nearly 60,000 leptospirosis-related deaths occur worldwide, resulting in 2.9 million Disability-adjusted Life Years (DALYs) each year [2]. Leptospirosis is endemic in most countries in South East Asia, including Malaysia. However, leptospirosis remains under-reported due to the wide range of clinical presentations associated with acute leptospiral infection [3, 4]. Human infections may be acquired through occupational, recreational, or environmental exposures, with direct contact with animals, soil, mud, or water at work placing individuals at risk.

There has been an increased trend in reporting leptospirosis cases in the last 10 years in Malaysia [5], where leptospirosis is considered an endemic disease, with sporadic outbreaks following rainy or flood seasons [6]. Previous literature indicated high seroprevalence and poor knowledge of leptospirosis and prevention practices among high-risk occupational groups [6–8], pointing to the need for the implementation of an effective intervention program in these groups.

Even though there are few studies that documented community perceptions of health and rodent-borne diseases [9] and protective practices against zoonotic infections among rural and slum communities [10], there have been limited studies which specifically focused on the knowledge, attitudes, beliefs and preventive behaviors towards leptospirosis. On the other hand, most studies [11–15] in various populations were shown to utilize questionnaires that were not properly developed and validated as well as some questionnaires with inadequate information on the validation processes [6, 12, 16]. The objective of the present study was to develop and validate a new knowledge, attitude, belief, and practice (KABP) questionnaire on leptospirosis among urban and rural populations in Malaysia. This questionnaire can serve as the baseline assessment in a community setting or as a tool for assessing the success of leptospirosis prevention and control initiatives in Malaysia or similar countries with leptospirosis endemicity.

## Methods

The development of the questionnaire and validation study took place in two phases. Phase 1 consisted of the questionnaire development stage, and phase 2 comprised validation studies, which included exploratory and confirmatory analyses.

### Phase 1: Questionnaire development

A thorough review of the literature was conducted to ascertain existing KABP, as well as to identify relevant items and scales in existing questionnaires on leptospirosis. To explore the level and scope of KABP on leptospirosis among local communities, eight focus group discussions (FGDs) were conducted among four urban and rural communities. Sixteen participants from rural areas were recruited through village leaders, and another 16 participants were selected from urban areas through social and professional networks of the research team. Interviews were conducted to explore their baseline knowledge of leptospirosis, its mode of transmission, and signs and symptoms. The interviews also explored their perceptions of the risk of contracting the infection and severity of the disease, as well as preventive aspects, including the use of personal protective equipment (PPE) and potentially useful health educational materials. The interviews were transcribed and analyzed using a thematic analysis. The findings from the FGDs on the depth of knowledge among the respondents were then used to develop relevant constructs for the questionnaire.

The first draft of the KABP questionnaire on leptospirosis in the Malay language was prepared by a panel of experts (an epidemiologist, an occupational health specialist, a microbiologist, a health educationist, and a medical statistician), complimented by the literature reviews and findings from the FGDs. This panel also helped in identifying and judging the content validity (relevance, coverage, and representativeness) of the items initially selected for inclusion in the questionnaire [17].

The questionnaire consisted of six sections, four of which encompassed items pertaining to knowledge, attitudes, beliefs, and practices, in addition to items on socio-demographics and residence. The participants provided information on age, gender, ethnic group or groups, household income, highest educational attainment, and years of similar work experience, as well as smoking status and common recreational activities. The questionnaire was designed as a self-administered questionnaire, according to standard protocols for questionnaire design and testing. The domains, concepts covered, and response options in the questionnaire are presented in Table 1.

**Table 1:** KABP questionnaire on leptospirosis

Sections	No. of Items	Concepts measured	Response options
General information	16	Socio-demographic, occupation, history of leptospirosis infection, smoking, recreational activities	Closed-ended, multiple-choice
Residence information	7	Distance from rivers/water sources, domestic animals, flood areas, waste disposal sites	Closed-ended, multiple-choice
Knowledge	24	Causes, exposure routes, symptoms and signs, detection methods, treatment, complications, prevention aspects	True/False/Don’t know; 1 = correct answer, 0 = wrong/don’t know
Attitudes	Validation 1		
	8 (3 reverse-scored items)	General attitude to leptospirosis	1 = Strongly disagree*,* 2 = Disagree*,* 3 = Not sure*,* 4 = Agree*,* 5 = Strongly agree
	Validation 2		
	13 (4 reverse-scored items)	Affect, behavior, and cognitive factors with regard to the prevention and treatment of leptospirosis and risk-related behaviors	1 = Strongly disagree*,* 2 = Disagree*,* 3 = Not sure*,* 4 = Agree*,* 5 = Strongly agree
Beliefs	Validation 1		
	4 (1 reverse-scored item)	General beliefs about leptospirosis	1 = Strongly disagree*,* 2 = Disagree*,* 3 = Not sure*,* 4 = Agree*,* 5 = Strongly agree
	Validation 2		
	21 (7 reverse-scored items)	Health beliefs about susceptibility, severity, benefits, barriers, and self-efficacy	1 = Strongly disagree*,* 2 = Disagree*,* 3 = Not sure*,* 4 = Agree*,* 5 = Strongly agree
Practices	Validation 1		
	12 (3 reverse-scored items)	Preventive and risk-reduction infection practices, including the use of PPE	1 = Never*,* 2 = Seldom*,* 3 = Often*,* 4 = Always
	Validation 2		
	19 (5 reverse-scored items)	Preventive and risk-reduction infection practices, including the use of PPE	1 = Never*,* 2 = Seldom*,* 3 = Often*,* 4 = Always*,* 9 = Not applicable

*KABP* knowledge, attitude, belief, and practice, *PPE* personal protective equipment

The questionnaire was then pre-tested with 10 participants (five each from the two FGDs) from urban and rural settings. The participants were recruited from two Malaysian states (Kelantan and Selangor) with a high reported incidence of leptospirosis. The FGDs served to test the face validity of the questionnaire and to determine how meaningful the concepts were to the studied community. After an open-ended discussion, the participants were asked to discuss and interpret each questionnaire item. The variability in their responses and their understanding of the questions, readability (layout and setting), and absence of ambiguity were evaluated. The results were used to produce a revised final version of the questionnaire, which was used in the remainder of the study.

### Phase 2: Validation studies

#### Validation study 1: Exploratory

The first part of the validation study was conducted from December 2015 to February 2016 to explore the psychometric properties of the questionnaire. In total, 214 participants were recruited through a multistage sampling method. This sampling strategy was carried out in the recruitment of adult respondents in the rural and urban communities of Kelantan, which was chosen because of its high leptospirosis incidence in Malaysia. The sampling procedures began with a list of districts stratified by urban and rural status followed by randomly selected two rural and two urban communities. The study was a household sample survey where the final sample unit was an adult per household who was eligible during the study period and randomly sampled for the survey. A total of 105 (49.1%) urban dwellers and 109 (50.9%) rural dwellers were selected. There was an equal male-female ratio, and the mean age was 43.4 (SD = 15.76) years. The majority (91, 42.5%) of the respondents had completed upper secondary school. The remainder held form six/certificate/diploma/higher degrees (50, 23.4%) or other lower educational levels (73, 34.1%).

The respondents were first briefed about the study. Informed consent was then obtained from the respondents who agreed to be involved in the study. The KABP leptospirosis questionnaire forms were given to each participant for self-administration.

The data analysis was performed in R version 3.3.2 [18], using the R Studio environment [19]. As the knowledge section consisted of unidimensional items with dichotomous responses, the knowledge section was analyzed by two-parameter logistic item response theory (2-PL IRT) analysis, using the ltm package version 1.0.0 [20]. Difficulty in the range of − 3 to + 3 and discrimination in the range of 0.35 to 2.5 were considered acceptable [21, 22]. Item fit was determined by the chi-square goodness-of-fit per item [22], and unidimensionality was determined by modified parallel analysis [23].

The attitude, belief, and practice sections were analyzed by exploratory factor analysis (EFA) using the psych package [24]. The principal axis factoring extraction method, with oblimin rotation was applied in the EFA. As the items in the attitude, belief, and practice sections had ordinal responses, these items were analyzed by EFA [25]. The items in each section were treated as continuous responses to allow evaluation of the dimensionality (number of factors) of the items [25]. To determine the number of extracted factors, eigenvalues > 1.0, parallel analysis, and scree plot inspection were performed [25]. Factor loadings > 0.4 were considered acceptable [26]. For internal consistency reliability, a Cronbach’s alpha coefficient > 0.65 was considered acceptable [27].

A sample size of 150 was required for an EFA study whenever 10 or more items were expected to have factor loadings of 0.4 [28]. The required sample size for 2-PL IRT followed the sample size for EFA because there are no definitive size for IRT, although it may range from 100 to 500 [29]. The sample size was inflated to 214 to account for 30% drop-out rate.

#### Validation study 2: Repeat EFA and confirmatory factor analysis (CFA)

In the second part of the validation study, which was conducted from July 2016 to January 2017, the revised KABP questionnaire was administered to adult respondents in urban and rural areas in Selangor to further explore and confirm the psychometric properties of the questionnaire.

In total, 759 respondents were recruited through a multistage sampling method. This sampling strategy was carried out in the recruitment of adult respondents in the rural and urban communities of Selangor, which was also chosen because of its high leptospirosis incidence in Malaysia. The sampling strategy was similar to that of Kelantan in the validation study 1. A total of 315 (41.5%) urban dwellers and 444 (58.5%) rural dwellers were involved. There were 384 (50.6%) male respondents and 375 (49.4%) female respondents, with a mean age of 35.2 (SD = 14.1) years. The majority of the respondents held form six/certificate/diploma/higher degrees (403, 53.1%). The remainder had completed upper secondary school (280, 36.9%) or other lower educational levels (76, 10.0%).

The exploratory sample consisted of 150 respondents: 62 (41.3%) from urban areas and 88 (58.7%) from rural areas. Of these, 79 (52.7%) were males, and 71 (47.3%) were females, with a mean age of 35.4 (SD = 14.4) years. The remaining 609 respondents were the confirmatory sample. This consisted of 253 (41.5%) urban and 356 (58.5%) rural respondents, of whom 305 (50.1%) were males, and 304 (49.9%) were females, with a mean age of 35.2 (SD = 14.1) years.

The methods for the 2-PL IRT analysis and EFA were similar to those described in validation study 1. The knowledge section was analyzed using 2-PL IRT and the whole sample in validation study 2. As the attitude and belief sections were revised following validation study 1, the sample was randomly split into exploratory and confirmatory samples for EFA and CFA, stratified by the location (urban vs. rural). This was achieved by splitting the full sample into urban and rural, followed by random sampling of the exploratory sample according to the strata size of the full sample (urba*n* = 150 × 41.5% = 62, rural = 150 × 58.5% = 88). The remaining 609 respondents were the confirmatory sample that had comparable urban-rural strata size.

The attitude and belief sections were analyzed by CFA using lavaan package version 0.5–22 [30]. The model fit assessment was based on the following fit indices and their respective cutoff values [25, 31]: χ2 *p* > 0.05, a comparative fit index (CFI) and Tucker-Lewis fit index (TLI) close to or more than 0.95, a root mean square error of approximation (RMSEA) ≤ 0.08, and a standardized root mean square residual (SRMR) ≤ 0.08. Raykov’s rho was used for the composite reliability [32] using the semTools package, version 0.4–14 [33]. A composite reliability value ≥0.7 was considered acceptable [34].

For EFA, a sample size of 150 is required whenever 10 or more items are expected to have factor loadings of 0.4 [28]. This was the sample size of the exploratory sample. For CFA, the minimum recommended sample size for is 200 because CFA typically requires large sample size whenever it involves complex models [35]), the remaining respondents were treated as the confirmatory sample.

## Results

### Questionnaire development and content and face validity

The concepts identified in the literature review on leptospirosis were very useful in the selection of items and formation of the relevant KABP sections in the questionnaire. The development of relevant constructs for inclusion in the questionnaire was further aided by the FGD sessions, which helped to identify additional items and local terminologies relating to leptospirosis that were meaningful to urban and rural communities.

In the content validation, the panel of experts judged the initial draft of the questionnaire. After a few revisions, the panel unanimously agreed that the included sections and items were consistent with the intended constructs in terms of relevance, coverage, and representativeness. For face validation, the questionnaire was pretested among urban and rural participants from two FGD sessions. According to their responses, after a few changes had been made to wordings, terminologies, and layout, most of the items were clear and easy to understand.

The final draft of the questionnaire at this stage contained 6 sections and 61 items (16 items on general information, 7 items on residence data, 24 items on knowledge, 8 items on attitude, 4 items on belief, and 12 items on practice.

### Validation study 1: Exploratory

As shown by the IRT analysis, the psychometric properties of the knowledge section were good (Table 2). With regard to the difficulty parameter, all the knowledge items were within or close to the acceptable range of − 3 to + 3. In terms of discrimination, most of the items were within the acceptable range. The K5i and K5iv items were slightly above the 2.5 cutoff value. K5iii exceeded the cutoff value by 4.2, and K5ii exceeded the cutoff by a large margin. However, in accordance with the advice of the experts, both K5ii and K5iii were retained because the content of these items was important. The item goodness-of-fit showed that nine of the items did not fit well (*p* <  0.05, Table 2). However, all these items were also retained in this section because they had acceptable difficulty and discrimination values. The amount of information tapped by the items between − 3 and + 3 difficulty range was 92.0%. The unidimensionality assumption was supported by the modified parallel analysis (*p* = 0.129). Cronbach’s alpha was 0.863, demonstrating internal consistency reliability.

**Table 2:** Results of the IRT analysis in the knowledge section of validation study 1 (*n* = 214)

Items	*b* (SE)	*a* (SE)	λ	χ^2^ (df = 8)	*P* values
K1 Penyakit kencing tikus juga dikenali sebagai penyakit leptospirosis (Rat urine disease is also known as leptospirosis)	−0.66 (0.19)	0.93 (0.21)	0.68	14.20	0.077
K2 Penyakit kencing tikus disebabkan oleh kuman (Rat urine disease is caused by germs)	−1.50 (0.25)	1.35 (0.29)	0.80	5.75	0.675
K3 Penyakit kencing tikus adalah penyakit haiwan yang boleh menjangkiti manusia (Rat urine disease is an animal disease that can infect humans)	−1.61 (0.32)	1.07 (0.25)	0.73	23.20	**0.003**
K4 Penyakit kencing tikus boleh dikesan melalui ujian darah (Rat urine disease can be detected through blood tests)	−1.80 (0.30)	1.40 (0.32)	0.81	7.75	0.459
K5 Kuman penyakit kencing tikus boleh memasuki badan manusia melalui: (Rat urine disease can enter the human body through:)					
K5i Luka pada anggota badan (wounds on limbs)	−0.03 (0.07)	3.05 (0.52)	0.95	14.62	0.067
K5ii Mata (eyes)	0.12 (1.64)	23.64 (330.54)	1.00	4.62	0.798
K5iii Hidung (nose)	0.10 (0.05)	6.70 (1.93)	0.99	16.18	**0.040**
K5iv Mulut (mouth)	−0.16 (0.07)	3.13 (0.56)	0.95	14.15	0.078
K5v Makanan yang tercemar (contaminated foods)	−1.48 (0.20)	1.98 (0.42)	0.89	17.07	**0.029**
K5vi Minuman yang tercemar (contaminated drinks)	−1.61 (0.23)	1.79 (0.39)	0.87	28.35	**< 0.001**
K5vii Bersalaman dengan pesakit penyakit kencing tikus (shaking hands with rat urine disease patient)	0.50 (0.46)	0.36 (0.16)	0.34	32.30	**< 0.001**
K6 Pesakit kencing tikus akan mengalami tanda-tanda berikut: (Rat urine disease patients will experience following symptoms:)					
K6i Demam (Fever)	−1.39 (0.18)	2.34 (0.51)	0.92	8.30	0.404
K6ii Sakit-sakit badan (Muscle Pains)	−1.27 (0.16)	2.30 (0.50)	0.92	6.95	0.542
K6iii Mata kuning (jaundis) (Yellow eyes (jaundice))	0.27 (0.16)	1.01 (0.21)	0.71	8.68	0.370
K7 Penyakit kencing tikus boleh mengakibatkan: (Rat urine disease patients may result in:)					
K7i Kematian (Death)	−2.96 (0.68)	1.59 (0.56)	0.85	4.07	0.851
K7ii Masalah pernafasan (Respiratory problems)	−0.65 (0.10)	2.42 (0.42)	0.92	22.06	**0.005**
K7iii Kegagalan buah pinggang (Kidney failure)	0.11 (0.10)	1.77 (0.30)	0.87	17.40	**0.026**
K7iv Kerosakan hati (Damage to the liver)	0.19 (0.10)	1.95 (0.33)	0.89	20.71	**0.008**
K8 Penyakit kencing tikus boleh dicegah dengan: (Rat urine disease can be prevented by:)					
K8i Pastikan persekitaran rumah bersih daripada sampah (Make sure home environment is clean from garbage)	−3.25 (0.89)	1.37 (0.53)	0.81	7.06	0.531
K8ii Elak diri dari mengharung air banjir (Avoid wading through floods)	−1.77 (0.31)	1.27 (0.29)	0.79	13.01	0.112
K8iii Jaga kebersihan diri (Keep personal hygiene)	−2.85 (0.69)	1.23 (0.40)	0.78	11.37	0.181
K8iv Minum air yang bersih (Drink clean water)	−3.29 (0.94)	1.26 (0.49)	0.78	19.34	**0.013**
K8v Pakai sarung tangan getah semasa bekerja (Wear rubber gloves at work)	− 1.95 (0.45)	0.86 (0.23)	0.65	12.79	0.119
K8vi Elak mandi di kawasan air terjun/lata/jeram/sungai/tasik/tali air yang tercemar (Avoid bathing in contaminated waterfalls/ rapids/rivers/lakes/waterways)	−2.79 (0.61)	1.46 (0.47)	0.83	10.55	0.229

*a* discrimination, *b* difficulty, *df* degree of freedom, *IRT* item response theory, *SE* standard error, χ^2^ chi-square, λ standardized loading

Items with *P* values < 0.05 in the assessment of the item fit are highlighted in bold

In the attitude section, the EFA suggested one factor solution. Six of eight items had acceptable factor loadings. Although this attitude factor with a reduced number of items had good reliability (Cronbach’s alpha = 0.76), the remaining items did not have good content coverage in relation to the attitude concept, thus required revision. In accordance with the tri-factor model, the attitude section consisted of affective, behavioral, and cognitive components relating to leptospirosis prevention and treatment and risk-related behaviors [17]. The number of items was increased from 8 to 13 in this section.

In the belief section, three of the original four items were retained. Although the remaining three items had good factor loadings, the belief factor had poor reliability (Cronbach’s alpha = 0.55). The latter was due to the small number of items. The small number of items might also indicate poor coverage of the belief concept. Based on the suggestions of the expert panel, this section underwent a major revision. In the revised version, the belief questions were based on the Health Belief Model, which is one of the most widely used conceptual frameworks for understanding health-related behaviors [36]. This model was utilized to explore beliefs about the susceptibility to leptospirosis infections and barriers to infections, in addition to the severity of infections and perceived benefits of disease prevention. It was also used to evaluate cues for actions and self-efficacy with regards to leptospirosis-related risks, treatment, and prevention, including the use of PPE. Subsequently, the number of items was increased from 4 to 21.

Finally, although the EFA of the items in the practice section suggested a two-factor solution, these factors could not be explained in term of the meaningful relationships between the items per factor. According to the results of the EEA, the content of P1 and P8i was redundant. Thus, this section underwent a major revision as suggested by the expert panel, and individual item scores were used instead of total factor scores to reveal specific practices. Thus, in the subsequent study (validation study 2), as described below, individual item scores were used, thus a factor analysis was not applied. Based on the experts’ opinion, the number of items was also increased from 12 to 19.

### Validation study 2: Repeat exploratory and confirmatory

The results of the IRT analysis in validation study 2 are presented in Table 3. Regarding the difficulty of each item, all the knowledge items were within the acceptable range of − 3 to + 3. For the discrimination parameter, most of the items were within the acceptable range. As K5iii and K5iv exceeded the cutoff by a small margin, these items were kept. The item fit showed that only one item showed a good fit to the model at α = 0.05 (K8vi, *p* = 0.060). However, all the items were retained because they had acceptable difficulty and discrimination values. The amount of information tapped by the items between − 3 and + 3 difficulty range was 93.1%. The unidimensionality assumption was not supported by the modified parallel test at α = 0.05 (*p* = 0.010). In terms of internal consistency reliability, Cronbach’s alpha was 0.867. A follow-up CFA (weighted least squares estimator) supported the unidimensionality assumption, based on a scaled CFI of 0.936 and a scaled TLI of 0.930, although the scaled RMSEA (0.163) indicated a poor model fit.

**Table 3:** Results of the IRT analysis in the knowledge section in validation study 2 (*n* = 759)

Items	*b* (SE)	*a* (SE)	λ	χ^2^ (df = 8)	*P* values
K1 Penyakit kencing tikus juga dikenali sebagai penyakit leptospirosis (Rat urine disease is also known as leptospirosis)	0.88 (0.16)	0.71 (0.1)	0.58	28.60	**< 0.001**
K2 Penyakit kencing tikus disebabkan oleh kuman (Rat urine disease is caused by germs)	−0.67 (0.09)	1.20 (0.13)	0.77	35.02	**< 0.001**
K3 Penyakit kencing tikus adalah penyakit haiwan yang boleh menjangkiti manusia (Rat urine disease is an animal disease that can infect humans)	−0.73 (0.09)	1.22 (0.13)	0.77	26.76	**0.001**
K4 Penyakit kencing tikus boleh dikesan melalui ujian darah (Rat urine disease can be detected through blood tests)	−0.37 (0.08)	1.17 (0.12)	0.76	16.23	**0.039**
K5 Kuman penyakit kencing tikus boleh memasuki badan manusia melalui: (Rat urine disease can enter the human body through:)					
K5i Luka pada anggota badan (wounds on limbs)	−0.04 (0.05)	2.11 (0.20)	0.90	35.63	**< 0.001**
K5ii Mata (eyes)	0.52 (0.04)	6.11 (0.74)	0.99	18.90	**0.015**
K5iii Hidung (nose)	0.38 (0.05)	6.45 (0.73)	0.99	28.73	**< 0.001**
K5iv Mulut (mouth)	0.15 (0.04)	5.14 (0.62)	0.98	36.24	**< 0.001**
K5v Makanan yang tercemar (contaminated foods)	−0.61 (0.06)	2.24 (0.24)	0.91	33.63	**< 0.001**
K5vi Minuman yang tercemar (contaminated drinks)	−0.63 (0.06)	2.30 (0.25)	0.92	38.73	**< 0.001**
K5vii Bersalaman dengan pesakit penyakit kencing tikus (shaking hands with rat urine disease patient)	0.85 (0.16)	0.69 (0.10)	0.57	46.87	**< 0.001**
K6 Pesakit kencing tikus akan mengalami tanda-tanda berikut: (Rat urine disease patients will experience following symptoms:)					
K6i Demam (Fever)	−1.07 (0.08)	2.19 (0.22)	0.91	38.43	**< 0.001**
K6ii Sakit-sakit badan (Pain)	−0.71 (0.09)	1.33 (0.13)	0.80	81.48	**< 0.001**
K6iii Mata kuning (jaundis) (Yellow eyes (jaundice))	0.52 (0.09)	1.17 (0.13)	0.76	33.82	**< 0.001**
K7 Penyakit kencing tikus boleh mengakibatkan: (Rat urine disease patients may result in:)					
K7i Kematian (Death)	−1.50 (0.14)	1.22 (0.14)	0.77	24.83	**0.002**
K7ii Masalah pernafasan (Respiratory problems)	0.32 (0.10)	0.85 (0.11)	0.65	47.94	**< 0.001**
K7iii Kegagalan buah pinggang (Kidney failure)	0.23 (0.10)	0.80 (0.11)	0.63	29.95	**< 0.001**
K7iv Kerosakan hati (Damage to the liver)	0.38 (0.11)	0.76 (0.10)	0.60	34.10	**< 0.001**
K8 Penyakit kencing tikus boleh dicegah dengan: (Rat urine disease can be prevented by:)					
K8i Pastikan persekitaran rumah bersih daripada sampah (Make sure home environment is clean from garbage)	−2.35 (0.32)	0.73 (0.11)	0.59	129.43	**< 0.001**
K8ii Elak diri dari mengharung air banjir (Avoid wading through floods)	−1.69 (0.26)	0.63 (0.10)	0.53	75.66	**< 0.001**
K8iii Jaga kebersihan diri (Keep personal hygiene)	−2.00 (0.26)	0.76 (0.11)	0.60	89.71	**< 0.001**
K8iv Minum air yang bersih (Drink clean water)	−2.30 (0.31)	0.74 (0.11)	0.59	91.10	**< 0.001**
K8v Pakai sarung tangan getah semasa bekerja (Wear rubber gloves at work)	−1.20 (0.20)	0.62 (0.09)	0.53	53.59	**< 0.001**
K8vi Elak mandi di kawasan air terjun/lata/jeram/sungai/tasik/tali air yang tercemar (Avoid bathing in contaminated waterfalls/ rapids/rivers/lakes/waterways)	−2.09 (0.22)	1.05 (0.13)	0.72	14.97	0.060

*a* discrimination, *b* difficulty, *df* degree of freedom, *IRT* item response theory, *SE* standard error, χ^2^ chi-square, λ standardized loading

Items with *P* values < 0.05 in the assessment of the item fit are highlighted in bold

In the EFA of the attitude section, the parallel analysis suggested four-factor solution, whereas the scree plot inspection suggested three-factor solution. The EFA was continued by fixing the number of factors to three, which corresponded to the tri-factor model of attitude. All the items in the Affect factor were grouped correctly in one factor. Four items in the Behavior factor and three items in the Cognitive factor were grouped together in one factor, thus this extracted factor was labeled as Behavioral-Cognitive factor. The third factor could not be interpreted. Thus, the EFA was repeated based on two factors, which successfully extracted interpretable two factors (Affect and Behavioral-Cognitive) based on the relationship between the items and the intended meaning of the factors (Table 4). All 13 items, which had standardized loadings ranging from 0.47 to 0.95, were kept. Both factors had acceptable internal consistency reliability.

**Table 4:** Results of the EFA and CFA of the attitude section in validation study 2

Factors	Items	EFA (*n* = 150)		CFA (*n* = 609)	
		λ	Reliability^a^	λ	Reliability^b^
Affect	A7 Saya tidak bimbang jika saya mengharung air banjir (I do not worry if I wade through flood water)	0.51	0.69	0.39	0.67
	A8 Saya tidak kisah jika ada tikus di persekitaran rumah saya (I do not mind if there are rats in my home environment)	0.86		0.77	
	A10 Saya tidak kisah jika persekitaran rumah saya kotor (I do not care if my home environment is dirty)	0.60		0.78	
	A13 Saya tidak bimbang walaupun tidak memakai alat lindung diri (kasut but, penutup muka dan sebagainya) semasa menguruskan sampah (I’m not worried even if I do not wear personal protective equipment (boots, face masks and similar tools) while handling garbage)	0.50		0.43	
Behaviour-Cognitive	A1 Saya akan pakai sarung tangan ketika menguruskan sampah (I will wear gloves when handling garbage)	0.54	0.90	0.45	0.85
	A2 Saya akan pastikan tong sampah sentiasa ditutup (I will make sure the garbage bin always be closed)	0.82		0.70	
	A3 Saya perlu bekerjasama dengan pihak kesihatan dalam aktiviti pencegahan dan kawalan penyakit kencing tikus (I have to cooperate with the health authorities in the prevention and control activities of rat urine disease)	0.89		0.81	
	A4 Saya akan pastikan keluarga saya membersih kawasan rumah (I will make sure my family clean up the house vicinity)	0.95		0.87	
	A5 Saya perlu maklumkan kepada pihak kesihatan sekiranya mendapat penyakit kencing tikus (I need to inform the health authorities if I got rat urine disease)	0.88		0.86	
	A6 Saya akan pastikan ahli keluarga tidak mandi di kawasan air terjun/lata/jeram/sungai/tasik/tali air yang tercemar (I will make sure the family members do not bath at contaminated waterfalls/ rapids/ rivers/lakes/waterways)	0.72		0.68	
	A9 Saya akan maklumkan kepada pihak kesihatan jika mengesyaki ada kes penyakit kencing tikus (I will inform the health authorities if I suspect there is a case of rat urine disease)	0.47		0.58	
	A11 Saya perlu berjumpa doktor sekiranya mengalami demam semasa wabak penyakit kencing tikus (I need to see a doctor if I have a fever during a rat urine disease outbreak)	0.63		0.64	
	A12 Saya perlu pakai alat lindung diri semasa menguruskan sampah (I need to use personal protective equipment while handling garbage)	0.50		0.46	

*EFA* exploratory factor analysis, *CFA* confirmatory factor analysis, λ factor loading/standardized loading

^a^Cronbach’s alpha, ^b^Raykov’s rho

The two-factor model was then tested by CFA. The CFA was performed using a robust maximum likelihood (ML) estimator because the data were not multivariate normal. As shown in Table 4, following the addition of two correlated errors (A1↔A2, *r* = 0.44; A11↔A12, *r* = 0.38), the model showed a good fit (χ^2^ [df = 62] = 262.51, *p* <  0.001; CFI_robust_ = 0.92; TLI_robust_ = 0.90; RMSEA_robust_ = 0.08; SRMR = 0.06). The correlation between the Affect factor and Behavioral-Cognitive factor was *r* = 0.36. The composite reliability of the Affect factor was slightly below the cutoff value of 0.7.

In the EFA of the belief section, the parallel analysis suggested six-factor solution, whereas the scree plot inspection suggested five-factor solution. The EFA was continued by fixing the number of factors to five, which corresponded to the five factors in the Health Belief Model. However, the items in these factors could not be interpreted. Thus, the EFA was repeated by iteratively removing 11 of the domain items based on standardized loadings and communalities. This resulted in a 10-item, five-factor solution, as displayed in Table 5. The Susceptibility factor was not extracted because none of the items in the proposed factor were factored together. The Self-efficacy factor was split into two sub-factors, which were Self-efficacy (environment) and Self-efficacy (personal). The internal consistency reliability was low for the Severity and Self-efficacy (personal) factors. The EFA derived five-factor model was then tested by CFA using a robust ML estimator because the data were not multivariate normal. The solution derived from the five-factor model was not valid because the data matrix was non-positive definite. The model could be fit only after the removal of the Severity and Self-efficacy (personal) factors, which resulted in a three-factor model (Table 5). The three-factor model showed a good fit, as shown in Tables 4 and 5 (χ^2^ [df = 6] = 31.49, *p* <  0.001; CFI_robust_ = 0.97; TLI_robust_ = 0.93; RMSEA_robust_ = 0.10; SRMR = 0.04). The correlations between the factors were: Benefits↔Barriers (*r* = 0.12); Benefits↔Self-efficacy (environment) (*r* = 0.69); Barriers↔Self-efficacy (environment) (*r* = 0.22). The composite reliability of the Benefits factor was far below the cutoff value of 0.7 (Raykov’s rho = 0.59), which was the result of the small number of items (only two) and low standardized loading for B14.

**Table 5:** EFA and CFA results for belief section in validation study 2

Factors	Items	EFA (n = 150)		CFA (n = 609)	
		λ	Reliability^a^	λ	Reliability^b^
Severity	B6 Saya percaya penyakit kencing tikus sukar diubati (I believe rat urine disease is difficult to treat)	0.62	0.37	–	–
	B19 Penyakit kencing tikus sukar dikenalpasti (Rat urine disease is difficult to be identified )	0.38		–	
Benefits	B14 Saya dapat elak penyakit kencing tikus dengan memakai alat lindung diri (I can avoid rat urine disease by wearing personal protective equipment)	0.51	0.60	0.72	0.59
	B16 Menjaga kebersihan persekitaran boleh mencegah pembiakan tikus (Maintaining the cleanliness of the environment can prevent the growth of rats)	0.81		0.58	
Barriers	B3 Saya tiada masa untuk membersihkan persekitaran rumah (I have no time to clean the home environment)	0.70	0.68	0.63	0.80
	B7 Saya utamakan kedai makan yang makanannya sedap berbanding kebersihan (I choose eateries because of tasty foods than cleanliness)	0.75		0.95	
Self-efficacy (environment)	B1 Saya yakin persekitaran bersih boleh mencegah penyakit kencing tikus (I believe the clean environment can prevent rat urine disease)	0.96	0.91	0.84	0.87
	B13 Saya tidak akan makan di tempat yang mempunyai kesan kehadiran tikus (I will not eat in places that have evidence of a rat infestation)	0.85		0.92	
Self-efficacy (personal)	B10 Saya yakin jika amalkan kebersihan diri, saya tidak akan dijangkiti penyakit kencing tikus (I am sure that if I have good personal hygiene, I will not be infected with rat urine disease)	0.34	0.50	–	–
	B18 Saya amat berhati-hati memilih kedai makan yang bersih (I am very careful in choosing clean eateries)	0.90		–	

*EFA* exploratory factor analysis, *CFA* confirmatory factor analysis, λ factor loading/standardized loading

^a^Cronbach’s alpha, ^b^Raykov’s rho

**Fig. 1:**
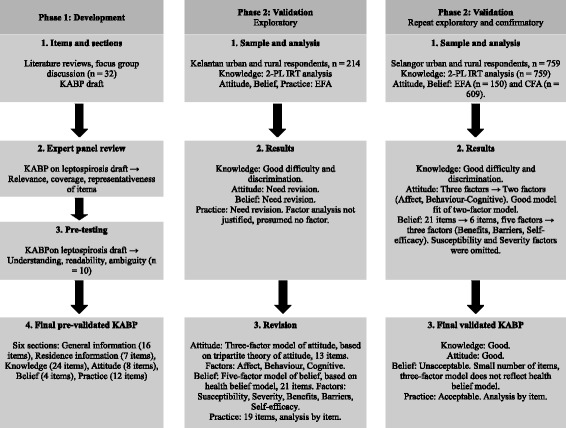
Summary of the development and validation stages of the questionnaire

A summary of the development and validation stages of the questionnaire is presented in Fig. 1.

## Discussion

The main aim of this study was to develop and validate a new KABP questionnaire on leptospirosis in Malaysia. Overall, the questionnaire was successful when applied to Malaysian urban and rural communities. Generally, the knowledge section showed good psychometric properties based on the difficulty and discriminatory parameters of the items. The analysis of the attitude section resulted in a good-fitting two-factor model, with good reliability. However, the analysis of the belief section showed low reliability for the Benefits factor and small number of items per factor, although the final three-factor model showed a good fit. The factor analytic approach was unsuitable for the practice section. The psychometric properties in this study could not be compared to previous studies on the knowledge, attitudes and practices on leptospirosis because of inadequate information and unclearly described development and validation processes in the studies [6, 13, 16, 11, 37].

Overall, based on the IRT analysis, the knowledge section showed good psychometric properties in the two validation studies. With regard to the difficulty parameter, all the difficulty values for the items were within or close to the acceptable range. For the discrimination parameter, the discrimination values for most of the items were within the acceptable range, except for items K5ii, K5iii, and K5iv, which exceeded the cutoff value of 2.5. However, these items were kept, given their importance in the assessment of knowledge about several important aspects of leptospirosis. In validation study 2, the IRT analysis showed that that only one item fitted the model at α = 0.05. This could be because the chi-square goodness-of-fit is sensitive to large sample sizes. As reported previously, as a sample size increases, small differences between observed and expected values can result in significant chi-square values [38]. In the present study, all the items had good difficulty and discrimination estimates, relatively small standard errors for estimates, and high standardized loadings (Table 3). Thus, all the items were retained. On the other hand, in validation study 1, the item goodness-of-fit showed that only nine of the items did not fit the model well. This problem was easily addressed because only two of the items had *p* values < 0.001, and the other seven items had an acceptable fit at α = 0.001.

In the attitude section, the initial items that were proposed had to be revised based on the findings of validation study 1, which showed poor content coverage. In validation study 2, the analysis resulted in a two-factor model of attitude (affect and behavioral-cognitive) instead of the proposed three-factor model (affect, behavior, and cognitive) [17]. In the context of behavioral and cognitive aspects of the prevention and treatment of leptospirosis and risk-related behaviors, thinking and actions are highly interrelated. For example, item A2 for behavior, “Saya akan pastikan tong sampah sentiasa ditutup” (“I will make sure that the waste basket is always closed”) contains both behavioral and cognitive components. Thus, the two-factor model of attitudes toward leptospirosis can be considered valid.

The belief section showed poor psychometric properties in both validation studies. The section was revised following the findings of validation study 1. Despite the revisions and the development of a good-fitting three-factor model after the CFA, only 6 of 21 items were retained in the belief section in validation study 2. The three-factor model also contradicted the five-factor Health Belief Model. As the number of items per factor was small in this section, the items may not have been representative of the intended factors. Therefore, the belief section should be comprehensively revised to develop representative items for Susceptibility, Severity, and Self-efficacy (personal) factors.

In the practice section, the initial plan was to employ the factor analytic method. However, the findings from validation study 1 showed that there were no interpretable correlations between the items. Thus, the scores for each item were utilized rather than the total scores for the section. An explanation about the type of practice was required for each item. These items reflected what the expert panel considered important preventive and risk-reduction infection practices in the community. Knowledge of these practices that are lacking in the assessed community is important to better plan effective intervention strategies.

The present study had a number of limitations. First, the participants were recruited only from Kelantan and Selangor, which represented the northeastern and western regions in Peninsular Malaysia, respectively. Cross-validation studies are needed in other parts of Peninsular Malaysia, as well as in Eastern Malaysia. Second, this study did not develop a satisfactory and valid belief section based on the Health Belief Model. To devise a valid measure of beliefs about leptospirosis, the belief section should undergo redevelopment and revalidation.

## Conclusion

In this study, a new Malay-validated KABP questionnaire was developed and validated among samples of urban and rural communities in Malaysia. The questionnaire consisted of 6 sections and 67 items (16 items on general information, 7 items on residence data, 8 items on knowledge, 13 items on attitude, 6 items on belief, and 17 items on practice). The knowledge and attitude sections were psychometrically valid based on IRT and factor analytic evidence. However, the psychometric properties of the belief section were unsatisfactory, despite being revised at the end of validation study 1. Further development of the belief section is warranted in future studies.

## Abbreviations

2-PL IRT: Two-parameter logistic item response theory
CFA: Confirmatory factor analysis
CFI: Comparative fit index
DALYs: Disability-adjusted Life Years
EFA: Exploratory factor analysis
FGD: Focus group discussions
HBM: Health Belief Model
IRT: Item response theory
KABP: Knowledge, attitude, belief, and practice
ML: Maximum likelihood
PPE: Personal protective equipment
RMSEA: Root mean square error of approximation
SD: Standard deviation
SRMR: Standardized root mean square residual
TLI: Tucker-Lewis fit index

## References

[1] Pappas G, Papadimitriou P, Siozopoulou V, Christou L, Akritidis N (2008). The globalization of leptospirosis: worldwide incidence trends. Int J Infect Dis.

[2] Torgerson PR, Hagan JE, Costa F, Calcagno J, Kane M, Martinez-Silveira MS (2015). Global burden of leptospirosis: estimated in terms of disability adjusted life years. PLoS Negl Trop Dis.

[3] Levett PN (2001). Leptospirosis. Clin Microbiol Rev.

[4] Victoriano AF, Smythe LD, Gloriani-Barzaga N, Cavinta LL, Kasai T, Limpakarnjanarat K (2009). Leptospirosis in the Asia Pacific region. BMC Infect Dis.

[5] MOH: Epidemiology and Current Situation of Leptospirosis in Malaysia. In. Putrajaya: Disease Control Division; 2015.

[6] Sulong MR, Shafei MN, Yaacob NA, Hassan H, Daud A, Zahiruddin WM (2012). Seroprevalence of Leptospirosis among Town Service Workers in Northeastern State of Malaysia. International Journal of Collaborative Research on Internal Medicine & Public Health.

[7] Sulong MR, Shafei MN, Yaacob NA, Hassan H, Daud A, Mohamad WMZW (2011). Risk factors associated with leptospirosis among town service workers. International medical. Journal.

[8] Sulong MR, Shafei MN, Yaacob NA, Hassan H, Daud A, Zahiruddin WM (2011). Town service Workers' knowledge, attitude and practice towards leptospirosis. Brunei Darussalam. Journal of Health.

[9] Salmón-Mulanovich G, Powell AR, Hartinger-Peña SM, Schwarz L, Bausch DG, Paz-Soldán VA (2016). Community perceptions of health and rodent-borne diseases along the inter-oceanic highway in Madre de Dios, Peru. BMC Public Health.

[10] Mason MR, Gonzalez M, Hodges JS, Munoz-Zanzi C (2015). Protective practices against zoonotic infections among rural and slum communities from south Central Chile. BMC Public Health.

[11] Abiayi E, Inabo H, Jatau E, Makinde A, Sar T, Ugbe D (2015). Knowledge, attitudes, risk factors and practices (KARP) that favor Leptospira infection among abattoir Workers in North Central Nigeria. Asian Journal of Epidemiology.

[12] Joseph A, Pedcris MO, November R, Hisako N, Yoshifumi T, Mitsuyasu Y (2016). Knowledge, attitude and practices towards leptospirosis among lakeshore communities of Calamba and Los Baños, Laguna, Philippines. Agriculture.

[13] Mohan AR, Chadee DD (2011). Knowledge, attitudes and practices of Trinidadian households regarding leptospirosis and related matters. International health.

[14] Navegantes de Araujo W, Finkmoore B, Ribeiro GS, Reis RB, Felzemburgh RD, Hagan JE (2013). Knowledge, attitudes, and practices related to leptospirosis among urban slum residents in Brazil. Am J Trop Med Hyg.

[15] Sakinah SNS, Suhailah S, Jamaluddin TZMT, Shafei MN, Malina O (2015). Seroprevalence of antibodies and knowledge, attitude and practices of leptospirosis to non high risk group in Selangor Leptospiral. International journal of public health and clinical. Sciences.

[16] Prabhu N, Meera J, Bharanidharan G, Natarajaseenivasan K, Ismail M, Uma A (2014). Knowledge, Attitude and Practice towards leptospirosis among municipal workers in Tiruchirapalli, India. International journal of pharma research and Health Sciences.

[17] Lawrence NK, Davis SF, Buskist W (2008). Attitudes and attitude change. 21st century psychology: a reference handbook.

[18] R Core Team: R: A language and environment for statistical computing. In. Vienna, Austria: R Foundation for Statistical Computing; 2016.

[19] R Studio Team (2016). RStudio: Integrated Development for R.

[20] Rizopoulos D (2006). Ltm: an R package for latent variable modelling and item response theory analyses. J Stat Softw.

[21] Aday L (1996). Designing and conducting health surveys.

[22] Baker FB: The basics of item response theory, 2nd edn. USA: ERIC Clearinghouse on Assessment and Evaluation; 2001.

[23] Drasgow F, Lissak R (1983). Modified parallel analysis: a procedure for examining the latent dimensionality of dichotomously scored item responses. J Appl Psychol.

[24] Revelle W (2016). Psych: procedures for personality and psychological research.

[25] Brown TA (2015). Confirmatory factor analysis for applied research.

[26] Stevens JP (2009). Applied multivariate statistics for the social sciences.

[27] DeVellis RF (2012). Scale development: theory and applications.

[28] Guadagnoli E, Velicer WF (1988). Relation to sample size to the stability of component patterns. Psychol Bull.

[29] Edelen MO, Reeve BB (2007). Applying item response theory (IRT) modeling to questionnaire development, evaluation, and refinement. Qual Life Res.

[30] Rosseel Y (2012). Lavaan: an R package for structural equation modeling. J Stat Softw.

[31] Schreiber JB, Nora A, Stage FK, Barlow EA, King J (2006). Reporting structural equation modeling and confirmatory factor analysis results: a review. J Educ Res.

[32] Raykov T (2001). Estimation of congeneric scale reliability using covariance structure analysis with nonlinear constraints. Br J Math Stat Psychol.

[33] Jorgensen TD, Pornprasertmanit S, Miller P, Schoemann A, Rosseel Y, Quick C et al: semTools: useful tools for structural equation modeling. R Package available on CRAN 2016.

[34] Hair JF, Black WC, Babin BJ, Anderson RE (2009). Multivariate data analysis.

[35] Kline RB (2011). Principles and practice of structural equation modeling.

[36] Croyle RT (2005). Theory at a glance: a guide for health promotion practice.

[37] Arbiol J, Borja M, Yabe M, Nomura H, Gloriani N, Yoshida S (2013). Valuing human leptospirosis prevention using the opportunity cost of labor. Int J Environ Res Public Health.

[38] Thompson B (1996). Research news and comment: AERA editorial policies regarding statistical significance testing: three suggested reforms. Educ Res.

